# Safety and Efficacy of Ondansetron and Simvastatin as Potential Adjunctive Treatment for Patients With Schizophrenia: A Systematic Review of Randomized Controlled Trials

**DOI:** 10.7759/cureus.40474

**Published:** 2023-06-15

**Authors:** Kokab Irfan Khan, Roba Al Shouli, Akhil Allakky, Asila A Ferguson, Aujala Irfan Khan, Baraa Abuzainah, Sai Dheeraj Gutlapalli, Dipabali Chaudhuri, Pousette Hamid

**Affiliations:** 1 Research, California Institute of Behavioral Neurosciences & Psychology, Fairfield, USA; 2 Pediatrics, California Institute of Behavioral Neurosciences & Psychology, Fairfield, USA; 3 Internal Medicine, California Institute of Behavioral Neurosciences & Psychology, Fairfield, USA; 4 Psychiatry, California Institute of Behavioral Neurosciences & Psychology, Fairfield, USA; 5 General Practice, California Institute of Behavioral Neurosciences & Psychology, Fairfield, USA; 6 Neurology, California Institute of Behavioral Neurosciences & Psychology, Fairfield, USA

**Keywords:** schizophrenia, icd-10, dsm-5, simvastatin, ondansetron

## Abstract

In a generation where advancements in research and understanding have led to remarkable achievements in medicine, it is still unfathomable that, after more than a century, the cause of schizophrenia is still a mystery. While antipsychotics, without a doubt, have brought on an exemplary revolution in the way psychiatric disorders are now treated, there are still imperative deficits that need to be addressed to ultimately enable individuals with schizophrenia to function normally in society. However, without a definite cause of schizophrenia, even though speculation has been made on its inflammatory and neurodegenerative nature, it has provided an unnecessary hindrance to finding further potential treatment modalities for these patients. Nevertheless, some trials are investigating potential adjunctive treatment regimens to antipsychotics, which can help achieve complete remission. Exploring these drugs will have significant implications for managing schizophrenia in future clinical practices. This systematic review was conducted between January 2012 to July 2022 according to Preferred Reporting Items for Systematic Review and Meta-Analysis guidelines to evaluate the safety and efficacy of ondansetron and simvastatin as adjunctive treatment to antipsychotics in adult patients with schizophrenia. This review included nine randomized controlled trials. Overall, both simvastatin and ondansetron, when used as adjunctive treatment in schizophrenia, appear to be safe. Ondansetron showed promising results, with all studies on this drug showing positive overall results on schizophrenia symptoms. On the other hand, simvastatin demonstrated mixed results, which can be attributed to the limited participants in the studies and the shorter duration of the trials. However, more extensive trials with uniform assessment tools are needed to demonstrate concrete evidence of the effectiveness of these drugs, whether alone or in combination with each other or perhaps another drug such as aspirin in schizophrenia.

## Introduction and background

Schizophrenia is a debilitating chronic mental disorder that affects around one in 300 people worldwide [1]. This number roughly equates to 24 million people around the world [1]. These individuals are two to three times more likely to die prematurely than the general population, attributed to the high mortality and morbidity of all psychiatric disorders [2-3]. Schizophrenia, which is diagnosed using the Diagnostic and Statistical Manual of Mental Disorders, fifth edition (DSM-5), or International Statistical Classification of Diseases and Related Health Problems, tenth revision (ICD-10), has symptoms that are divided into positive (hallucinations, delusions, disorganized speech, and behavior), negative (anhedonia, avolition, blunted affect, alogia, and asociality), and cognitive deficits (memory issues, impaired sensory perception, and inability to process social cues) [4-5]. For a DSM-5 diagnosis of schizophrenia, at least two or more symptoms must be present for a significant amount of time over a one-month period, and signs of disturbance should be continuously present for at least six months [4]. For more than a decade, the primary treatment of schizophrenia has been antipsychotics (first and/or second generation). These drugs have an incredible effect on positive symptoms, but their effect on negative symptoms is inadequate [6]. Therefore, the discovery of treatment directed toward negative symptoms is of utmost importance as these symptoms are indicative of individual functionality in society, and impairment arising from schizophrenia accounts for a large part of long-term morbidity and poor functional outcomes in these patients [7-8].

A century after its discovery, the cause of schizophrenia remains unknown. Schizophrenia is thought to be a complex interplay between genetic and environmental predisposing factors that affect brain function by impairing cognition, processing, and interpretation of stimuli and experiences. However, growing evidence suggests it might also be a potential inflammatory disease, with affected individuals having elevated inflammatory markers, immune cell counts, and antibody titers in their blood, cerebrospinal fluid, and central nervous system [9-12]. 

Simvastatin, a lipid-lowering drug, is a hydroxymethylglutaryl-coenzyme A reductase inhibitor. Although the effect of statins on schizophrenia is debatable, a recent analysis found that their utilization improved psychiatric symptoms [13-14]. Moreover, studies have recently highlighted the anti-inflammatory properties of statins and their ability to decrease C-reactive protein (CRP) [15]. In a meta-analysis, CRP levels were found to increase in schizophrenia despite using antipsychotics [16]. In addition, some studies have shown an association between CRP levels and higher scores on PANSS (Positive and Negative Syndrome Scale) negative symptoms [17-18]. 

Serotonin 5-hydroxytryptamine-3 (5-HT3) receptors are key players in the development of cognitive dysfunction in schizophrenic patients [19]. Ondansetron, a selective serotonin (5-HT3) receptor antagonist, is approved to treat drug-induced nausea and vomiting. In recent years, there has been growing speculation about using ondansetron as a potential adjunctive treatment for cognitive impairment in schizophrenia, but it remains controversial. 5-HT3 receptor antagonism is hypothesized to be a contributing factor in the therapeutic advantages of atypical antipsychotic agents by providing a beneficial mix of dopaminergic and 5-HT3 blockade [20-21]. Furthermore, animal studies have found ondansetron to modulate mesolimbic dopamine activity without affecting striatal dopamine systems [22]. A recent meta-analysis found that adjunctive ondansetron is safe and efficient for schizophrenia by improving negative and general psychopathology symptoms [23]. Additionally, there is proof that 5-HT3 antagonists can reduce inflammation in human monocytes by inhibiting secretions of tumor necrosis factor alpha or interleukin-1beta [24].

This systematic review aims to explore the effect of simvastatin and ondansetron on schizophrenic patients, specifically their efficacy and safety. There are several studies done comparing the efficacy and safety of both these drugs individually. Moreover, a multi-center trial of six months studied the combination of ondansetron and simvastatin, and they found that the interaction between ondansetron and simvastatin was significant (p=0.006) at three and six months, where both drugs appeared to improve negative symptoms [25]. On other assessments, this combination demonstrated significance as well; the total PANSS scores were significantly improved; Calgary Depression Scale for Schizophrenia (CDSS), p=0.001; Clinical Global Impression (CGI), p=0.04; and EuroQol-5 Dimension (EQ-5D), p=0.02 [25]. Hence, we aimed to evaluate nine randomized controlled trials (RCTs) for the safety and efficacy of both these drugs for schizophrenia. Furthermore, understanding the effectiveness and tolerability of these medications will further guide combination adjunct therapy to treatment as usual and substantially impact the global and economic burden of schizophrenia.

## Review

Methodology

For this systematic review, we used the Preferred Reporting Items for Systematic Review and Meta-Analysis (PRISMA) 2020 guidelines [26].

Search Sources and Strategy

The study was executed using a pre-specified search strategy with strict eligibility criteria. First, an extensive search of literature published between 2012 and 2022 of databases such as PubMed, PubMed Central (PMC), ScienceDirect, and Google Scholar was completed to retrieve relevant literature that reported ondansetron or simvastatin and their response or outcome in schizophrenic patients. A combination of search terms used was “ondansetron,” “simvastatin,” and “schizophrenia” in all fields. Reference lists from the articles were also scrutinized for appropriate supplementary studies.

Eligibility Criteria and Screening

EndNote (Clarivate 1.9, Philadelphia, United States) was used to filter for and eliminate duplicates. The remaining papers were then screened based on their titles and abstracts. The quality of the paper was then used to evaluate the complete text of the results. Only papers that met >70% of the quality appraisal’s assessment criteria were included. Only articles published in the English language between 2012 and 2022 were included in the list. The adult population of humans (those older than 18) was also a focus. This evaluation did not include papers involving the pediatric population, grey literature, and animal research. The Cochrane Bias Assessment tool was used for the screening as all the studies were RCTs and only those with sound methodological quality were included.

Results

**Figure 1:**
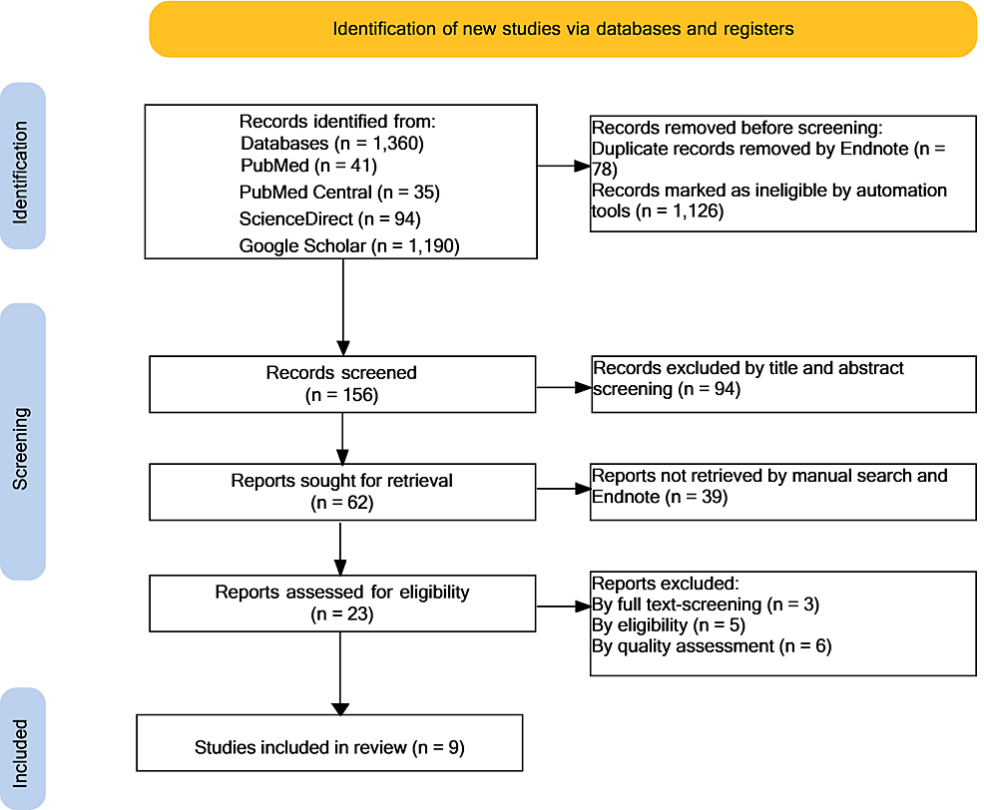
PRISMA flowchart PRISMA: Preferred Reporting Items for Systematic Reviews and Meta-Analyses, n: number of records

A total of 1,360 results were identified from the databases mentioned above; 78 results were removed because of duplicates and automation tools (1,126); and 156 were screened. Screening titles and abstracts excluded 94. Moreover, the remaining 62 were sought for retrieval, but 39 could not be retrieved; 23 reports were evaluated for eligibility; and 14 were removed after screening for full text, English language-only papers, and quality assessment. The PRISMA flowchart in Figure 1 demonstrates the filtering process: The final criteria were met by nine articles.

Below is a summary of the outcomes and characteristics of the RCTs included in this systematic review Table 1.

**Table 1:** Brief overview of the studies mentioned in this systematic review SSD: Schizophrenia Spectrum Disorders, SAE: Serious Adverse Event, 5-HT3: 5-hydroxytryptamine 3, PANSS: Positive and Negative Syndrome Scale, TAU: Treatment as usual, CGI: Clinical Global Impressions, GAF: Global Assessment of Functioning, AIMS: Abnormal Involuntary Movement Scale

Author and Year of Publication	Purpose of study	Duration of study	Location	Number of patients	Conclusion
Pourghasem et al., 2022 [27]	To assess the effects of aspirin and simvastatin, used as adjunctive treatment to placebo in individuals with schizophrenia, on general psychopathology and their negative and positive symptoms.	8 weeks	Iran	45	Demonstrated that simvastatin and aspirin could lessen the negative, general psychopathology, and positive symptoms. However, the effectiveness of both drugs was similar, and it is advised to use these medications as an adjunct, not the primary treatment.
Ashrafi et al., 2021 [28]	To assess the effects of simvastatin and schizophrenia symptoms.	6 weeks	Iran	40	Could not significantly reduce the symptoms of these patients. Although the result is negative for any effect, this study is worth considering as its data can be included in potential future meta-analyses in the field.
Kulkarni et al., 2021 [29]	To compare adjunctive ondansetron or placebo to atypical antipsychotics for people who have schizophrenia.	12 weeks	Australia	81	This treatment trial provides some evidence for adjunctive ondansetron medication on cognitive symptoms of schizophrenia.
Sommer et al., 2021 [30]	If simvastatin augmentation can help patients with early SSD symptoms and cognition.	12 months	Netherlands	119	No significant difference between simvastatin treatment and placebo in terms of the overall severity of symptoms at 12 months. After six months and 24 months of follow-up, the simvastatin group experienced much less severe symptoms. No significant treatment impact was discovered for secondary outcomes or cognition. Compared to simvastatin, SAEs happened more frequently with a placebo (19%).
Mubeen et al., 2018 [31]	To evaluate the effects of ondansetron on the negative and depressive symptoms of patients with schizophrenia.	12 weeks	Pakistan	60	Ondansetron improved negative symptoms and cognitive function. It might help reduce symptoms of schizophrenia. The study findings support the notion that ondansetron can be effective in addition to conventional medication, particularly for negative symptoms.
Samadi et al., 2017 [32]	This study examined whether risperidone combined with ondansetron helped lessen negative and depressive symptoms in people with treatment-resistant schizophrenia.	12 weeks	Iran	38	Proved that, when ondansetron is used as an adjunct, it reduces negative symptoms in patients with schizophrenia and can be used as adjunctive medication for negative symptoms and cognitive impairments.
Tajik-Esmaeeli et al., 2017 [33]	To assess the effects of simvastatin as an adjunct on the negative symptoms of schizophrenia.	8 weeks	Iran	66	Simvastatin was found to have a beneficial effect on the negative symptoms experienced by schizophrenia patients; however, further research is required to corroborate these findings.
Hema et al., 2016 [34]	To evaluate the effectiveness of the 5-HT3 receptor antagonist ondansetron as an adjuvant in reducing psychopathology in schizophrenia.	12 weeks	India	25	The result of this study is in line with earlier research that has been mentioned in the literature. Remarkably, unfavorable symptoms have significantly improved since baseline.
Chaudhry et al., 2014 [35]	To investigate the effects of simvastatin and ondansetron as adjunctive treatments on positive, negative, and general psychopathology.	12 weeks	Pakistan	36	Simvastatin and ondansetron show evidence of symptom reduction on the PANSS total score compared to TAU, although this was not statistically significant. On CGI, GAF, and AIMS, no appreciable changes were discovered in the secondary analyses.

Discussion

In this systematic review, we analyzed nine studies. We divided them into two groups according to drug type to analyze the safety and efficacy of both drugs separately, and one study compared the efficacy and safety of both drugs in the same trial. We aimed to analyze and then assess the effect of ondansetron and simvastatin on the PANSS, which is used to measure the severity of schizophrenia; hence, we sought to explore the effect on total scores as well as on its subscales (positive symptom, negative symptom, and general psychopathology), cognition by various scales (scales varied with different trials), and some specific scales for negative symptoms that helped us identify the specific symptoms affected. Additionally, we addressed the safety profiles of these drugs. We also attempted to gauge additional results on depressive symptoms.

Below is a summary of the various scales used in each RCT in this systematic review Table 2. 

**Table 2:** Scales used in the randomized controlled trials x = Not Assessed, √ = Assessed AIMS: Abnormal Involuntary Movement Scale, BACS: Brief Assessment of Cognition in Schizophrenia, CDSS: Calgary Depression Scale for Schizophrenia, CGI/CGI-S: Clinical Global Impressions Scale/ Clinical Global Impressions-Severity Scale, ESRS: Extrapyramidal Symptoms Rating Scale, GAF: Global Assessment of Functioning, HRSD/HDRS: Hamilton Rating Scale for Depression/ Hamilton Depression Rating Scale, MADRS: Montgomery–Åsberg Depression Rating Scale, MCCB: MATRICS™ Consensus Cognitive Battery, PANSS: Positive and Negative Syndrome Scale, WAIS/WAIS-R: Wechsler Adult Intelligence Scale/ Wechsler Adult Intelligence Scale-Revised

Author and Year of Publication	AIMS	BACS	CDSS	CGI/CGI-S	ESRS	GAF	HRSD/HDRS	MADRS	MCCB	PANSS	WAIS/WAIS-R
Pourghasem et al., 2022 [27]	x	x	x	x	x	x	x	x	x	√	x
Ashrafi et al., 2021 [28]	x	x	x	x	x	x	x	x	x	√	x
Kulkarni et al., 2021 [29]	√	x	x	x	x	x	x	√	√	√	x
Sommer et al., 2021 [30]	x	√	√	x	x	√	x	x	x	√	x
Mubeen et al., 2018 [31]	x	x	x	x	x	x	√	x	x	√	√
Samadi et al., 2017 [32]	x	x	x	x	x	x	x	x	x	√	√
Tajik-Esmaeeli et al., 2017 [33]	x	x	x	x	√	x	√	x	x	√	x
Hema et al., 2016 [34]	x	x	x	√	x	x	x	x	x	√	x
Chaudhry et al., 2014 [35]	√	x	x	√	x	√	x	x	x	√	x

Efficacy of Ondansetron

We analyzed five studies out of nine to assess the efficacy of ondansetron.

In 2021, Kulkarni et al. studied 81 patients for 12 weeks with either schizophrenia or schizoaffective disorder [29]. A total of 41 patients received ondansetron 8 mg/day: two were on paliperidone, seven were on risperidone, nine were on olanzapine, 10 were on clozapine, five were on aripiprazole, two were on clopixol/zuclopenthixol, and it was unclear what antipsychotics the six remaining patients were on [29]. A total of 40 patients received a placebo: one was on paliperidone, five were on risperidone, 13 on olanzapine, 11 on clozapine, five were on aripiprazole, two on clopixol/zuclopenthixol, and it was not reported what antipsychotics the remaining three patients were on [29]. It was unclear which antipsychotic had the most significant effect with ondansetron as an adjunct. Ondansetron usage showed marginal significance in the total PANSS (p=0.06) by week 12, with the cognitive subscale showing marked improvement (p<0.05) [29]. However, no difference was found in the rest of the PANSS subscales [29]. On Montgomery-Åsberg Depression Rating Scale, no difference was noted between both the groups (p>0.5) [29]. MATRICS Consensus Cognitive Battery showed no difference as well [29]. However, it was found that, on the Abnormal Involuntary Movement Scale (AIMS), ondansetron improved tardive dyskinesia [29]. Moreover, the study included both schizophrenia and schizoaffective patients; however, it was unclear which disorder was affected more when using ondansetron as an adjuvant.

On the other hand, a study done in 2018 by Mubeen et al. on 60 patients (48 patients completed the trial) for 12 weeks found a significant difference in negative symptoms (p=0.002) using PANSS between the experimental (ondansetron (4-8 mg/day) and risperidone (4-6 mg/day)) and the placebo (placebo and risperidone) groups [31]. They further assessed the effect of ondansetron by Wilcoxon test before and after treatment of the interventional group and found significant improvement in all the subsets: blunted affect (p<0.002), emotional withdrawal (p<0.003), poor rapport (p<0.001), passive/apathetic social withdrawal (p<0.005), difficulty in abstract thinking (p=0.001), and stereotyped thinking (p<0.001) [31]. However, the only negative symptom with no significant difference was a lack of spontaneity and flow of conversation [31]. Mubeen et al. also investigated the effect of ondansetron on depressive symptoms, and hence Hamilton Rating Scale for Depression (HRSD) usage showed no significant difference compared to the placebo group (effect size=0.15) [31]. Additionally, there was a significant difference noted on the Wechsler Adult Intelligence Scale-Revised (WAIS-R) for comprehension and object assembly subscales [31]. Based on the Wechsler Adult Intelligence Scale (WAIS) subsets, ondansetron improved visual memory significantly [31].

Similarly, in 2017, Samadi et al. studied 38 patients for 12 weeks in a double-blind trial [32]. Eighteen patients in the experimental group received ondansetron 4 mg/day in the first week, which was increased to 8 mg in the second week, plus risperidone (8 mg/day) [32]. This group was associated with significant improvement (p<0.001) at two consecutive points in the total scores of the PANSS and subscale for negative symptoms as well as cognition compared to the placebo group [32]. Hence, ondansetron was more effective on negative symptoms than on positive symptoms on the PANSS [32]. The most significant effect was on passive/apathetic social withdrawal on the PANSS [32]. On Wilcoxon, significant differences exist, except for spontaneity and flow of conversation [32]. The WAIS-R also demonstrated significant differences between both groups [32]. Object assembly and comprehension subscale specifically showed significant differences (p<0.001) between the two groups after treatment [32]. Additionally, visual memory, another subset of WAIS, showed a difference (p<0.05) [32]. However, it was noted that ondansetron did not affect depressive symptoms (effect size=0.13) [32].

Furthermore, in 2016, Hema et al. observed 25 patients for 12 weeks in an open-label feasibility study and found that adding ondansetron as an adjuvant to treatment as usual (clozapine (466±264 mg) or amisulpride (222±56.5 mg)), and increasing dose of ondansetron from 8 mg/day to 16 mg/day, resulted in statistically significant improvement (p<0.0001) in the total PANSS scores, including all subscale domains (positive, negative, and general psychopathology) as well on the Clinical Global Impression-Severity Scale (CGI-S) [34]. 

Additionally, only one study out of nine discussed both simvastatin and ondansetron. Chaudhry et al., in a 12-week feasibility study, studied 36 patients to assess the effect of ondansetron and simvastatin [35]. The control group received treatment as usual (TAU); the second group received TAU plus simvastatin (20 mg/d increased to 40 mg/d after four weeks), and the third group was given TAU plus ondansetron (8 mg/d) [35]. A more significant reduction in symptoms and the total PANSS scores compared to TAU was noted in both ondansetron and simvastatin groups [35]. However, it was not statistically significant and was mainly seen in positive and general psychopathology subscales [35]. There appears to be limited effect on negative symptoms [35]. No difference was found on the CGI between the three groups [35]. However, on the Global Assessment of Functioning (GAF) scale, there was less improvement in the ondansetron and TAU groups compared to the simvastatin group [35]. On AIMS, both simvastatin and ondansetron appear to be well-tolerated [35].

Safety of Ondansetron

To assess the safety profile of ondansetron, we analyzed the studies for its tolerability and adverse events. Kulkarni and colleagues demonstrated that ondansetron was well tolerated, and only four patients suffered constipation, which was so severe that they had to drop out of the study [29]. Moreover, Mubeen et al. found that, out of 30 patients, a majority (19) reported adverse events; seven reported constipation, five reported restlessness and insomnia, and seven had exhaustion, confusion, and nausea [31]. Similarly, Samadi and colleagues reported that 11 out of 18 patients had adverse events: two had insomnia and restlessness, two experienced constipation, and rest reported confusion, nausea, and exhaustion [32]. Meanwhile, Hema et al. stated no major side effects [34]. Furthermore, no safety issues were reported regarding prolonged QTC intervals due to the use of ondansetron and the respective antipsychotics used in these studies.

Efficacy of Simvastatin

To assess the efficacy of simvastatin, we analyzed five studies out of nine.

In 2022, Pourghasem et al. studied 45 patients for eight weeks and divided them into two interventional groups: one received risperidone (4 mg/day) and aspirin (325 mg/day, twice daily) and the other group received risperidone and simvastatin (40 mg/day) and one control group received risperidone and placebo [27]. Statistically significant scores were found on the total PANSS (p<0.001) in both groups compared to that of the placebo group. In addition, the positive symptom subscale of the PANSS significantly decreased in groups treated with aspirin (p=0.006) and simvastatin (p=0.005) [27]. Moreover, the intervention groups demonstrated a noteworthy score on negative symptoms (p<0.001) as well as on general symptoms of the PANSS (p<0.001) [27]. No significant difference between the groups receiving aspirin and simvastatin was found in the total PANSS and its subscales (positive, negative, and general psychopathology) [27]. This indicates that aspirin and simvastatin may have a similar degree of effect on schizophrenia, with none being superior to the other. 

On the contrary, in 2021, Ashrafi and colleagues conducted a trial of 40 patients for six weeks, of which each group (20 patients) received simvastatin (40 mg/day) or placebo and were on atypical antipsychotics: chlorpromazine (200-1000 mg/day) plus, if needed, bipyridine (2-6 mg/day) and/or clonazepam (0.5-2 mg/day) [28]. They found that, although there was a significant reduction in scores on the positive and negative subscales of the PANSS, it was, however, statistically not significant compared to the placebo (p>0.05) [28]. This could be attributed to the shorter duration of the study.

However, Sommer et al. studied 119 patients with schizophrenia spectrum disorder (SSD) for 12 months [30] who either received simvastatin 40 mg/d or a placebo in addition to TAU. On the PANSS, the total score showed no effect after 12 months, and treatment x time interaction was substantial (p=0.025), indicating that the group difference in symptom severity varied over time [30]. However, it revealed a significantly lower score at six months (mean difference=-4.8; p=0.021; 95% CI:-8.8 to -0.7) and 24 months (mean difference=-4.7; p=0.040; 95% CI:-9.3 to -0.2) [30]. On a Brief Assessment of Cognition in Schizophrenia (BACS), the intervention group did not differ from the placebo in cognitive performance [30]. However, the treatment x time interaction was significant for the PANSS general sub-scores (p=0.017), which was lower in the experimental group at six months (mean difference=-2.7, p=0.017, 95% CI:-4.9 to -0.5) [30]. The main treatment effect on the GAF scale was not statistically significant [30]. However, a significant treatment x time interaction was noted (p=0.052), with higher general functioning noted in the interventional group at 24 months (mean difference=4.9, p=0.048, 95% CI:0.04-9.8) [30]. On the CDSS, the interventional group was noted to have significantly lower depressive symptoms than the placebo group at six months (p=0.021) [30]. Overall, this study found that simvastatin augmentation is not beneficial for symptom severity and cognition in SSD. These findings can be attributed to a lack of treatment adherence and possible induction of the ceiling effect in cognition as a majority of the participants in this RCT had relatively high education and as a result showed minimal cognitive deficits [30]. Additionally, the study included participants with SSD (i.e. those with schizophrenia, schizoaffective, schizophreniform, or psychotic disorder not otherwise specified); however, it was unclear which disorder was affected more significantly when using simvastatin as an adjuvant.

Moreover, in 2016, Tajik-Esmaeeli and colleagues observed 66 patients, of which 33 received simvastatin (40 mg/day) and risperidone (4-6 mg/day) and the rest half received a placebo and risperidone (4-6 mg/day) for eight weeks [33]. They noted baseline negative symptoms scores were higher than positive in both groups [33]. However, the intervention group demonstrated a noteworthy reduction in the negative symptoms’ subscale of the PANSS (p=0.003), and the time x treatment interaction was noteworthy in comparison with the groups (p=0.001) [33]. A similar difference was noted for the total scores of the PANSS (p=0.001) and time x treatment interaction (p<0.001) [33]. However, positive and general psychopathology results were insignificant on the PANSS [33]. On the Hamilton Depression Rating Scale (HDRS) and Extrapyramidal Symptoms Rating Scale (ESRS), no significant difference was noted [33].

Furthermore, Chaudhry et al. found on GAF that there was more improvement in the simvastatin group than in the ondansetron and TAU groups [35]. They also demonstrated an improvement in symptoms for both the ondansetron and simvastatin groups compared to the TAU group, especially seen in positive and general psychopathology on PANSS subscales [35]. However, it was not statistically significant and can be attributed to the small size and duration of the study. This should be investigated by future trials of longer duration and size.

Safety of Simvastatin

Ashrafi et al. had three cases that suffered from tremors, although it was not statistically significant compared to the placebo group (p>0.05) [28]. Moreover, Sommer and colleagues found that statins were safe and generally well-tolerated as more side effects (myalgia and rhabdomyolysis) occurred 19% more in the placebo group than in the interventional group [30]. Finally, the Tajik-Esmaeeli et al. study had some patients who suffered from extrapyramidal symptoms (sleep disturbances, dystonia, and akathisia); two had increased appetite, two had dry mouth, three had constipation, two had drowsiness (daytime), five had fatigue, four had a headache, and five experienced insomnia [33].

Limitations

We had several restrictions apart from our inclusion criteria between 2012 and 2022 and English literature. We also only aimed to evaluate RCTs. Hence, this review did not include a meta-analysis or systematic review, which could have increased the subset of patients included in this review and given us an insight into the prior years. Additionally, two additional multi-center studies with a good subset of patients were excluded from this review as they were only conference abstracts and not full-text articles. Those two studies evaluated the efficacy of the combined administration of ondansetron and simvastatin as an adjunct treatment for schizophrenia. Another disadvantage was the scales used since there is no gold standard to interpret the results of the PANSS and other instruments. Clinicians must rely on experience with individual populations to interpret these scores, which may change the clinical significance of these scores. Moreover, there is no universal standard scale used. As such, the studies used a variety of scales, which could result in diverse conclusions and negate the significance of these trials compared to other studies. The studies included in this review also all had various antipsychotic regimens, which could have arguably led to varying results as the potential interaction with each antipsychotic differs. Furthermore, there is a possibility of potential publication bias in this review, as studies with negative findings may be less likely to be published. Hence, we recommend additional large multi-center trials with longer duration to conclusively detect a treatment effect of ondansetron and simvastatin since all the RCTs included in this review had a low number of participants and were carried out over short durations. Moreover, a detailed analysis of the heterogeneity of study populations, including age, gender, and disease severity, could provide valuable insight into potential moderators of the treatment response.

## Conclusions

Our study contained nine RCTs; most were double-blind studies. Individually, both drugs appear to be effective for the treatment of schizophrenia, even though we did notice a variance in the results for simvastatin and ondansetron. Generally, both drugs were well-tolerated. However, caution must be considered before conclusively categorizing these drugs as adjunct therapies. Several of the RCTs we analyzed had small sample sizes, potentially limiting the findings’ validity. Another possibility of the variance in the results could be due to the adherence to the medication, which could have led to optimal results not being achieved. Even though we aimed only to evaluate simvastatin and ondansetron in relation to schizophrenia, literature may exist that includes the same patient profile without mentioning it, resulting in the low sensitivity of this review despite our search strategy. The future of combination therapy is promising, but developing efficient treatment has proven challenging and slow. Another substantial issue is establishing and putting into practice an effective treatment plan that is safe, accessible, and well-tolerated. Therefore, our study is a foundation for additional multi-center trials to compare ondansetron and simvastatin combinations with longer durations. These drugs are readily available and can potentially be repurposed as adjunct treatments in psychiatric disorders. Moreover, we suggest it is vital to analyze whether simvastatin and ondansetron cause a synergistic, antagonistic, or additive effect when combined with each other, as well as other antipsychotic and anti-inflammatory drugs. Furthermore, studies should be carried out in the future to explore the safety of these drugs for long-term use in schizophrenia. Finally, we suggest researchers investigate a universal interpretation guideline for scales such as the PANSS, allowing results from future studies to be consistent and easily comparable, leading to faster implementation worldwide.
